# Pyopneumothorax Secondary to Pulmonary Tuberculosis Superadded by Congenital Factor XIII Deficiency: A Case Report

**DOI:** 10.7759/cureus.47350

**Published:** 2023-10-19

**Authors:** Jyoti Bajpai, Jay Tewari, Shubhajeet Roy, Ajay K Verma, Shailendra P Verma, Surya Kant

**Affiliations:** 1 Respiratory Medicine, King George's Medical University, Lucknow, IND; 2 Medical Sciences, King George's Medical University, Lucknow, IND; 3 Respiratory Medicine, Pulmonary Critical Care Medicine, King George's Medical University, Lucknow, IND; 4 Clinical Hematology, King George's Medical University, Lucknow, IND

**Keywords:** congenital disease, autosomal inheritance, factor xiii deficiency, tuberculosis, pyopneumothorax

## Abstract

Pyopneumothorax is a rare complication of pulmonary tuberculosis, contributing significantly to morbidity and mortality. Additionally, factor XIII deficiency, a rare bleeding disorder, may pose a diagnostic challenge due to normal results in routine coagulation tests. We present the case of an 18-year-old boy who presented with a history of left-sided pyopneumothorax secondary to drug-sensitive *Mycobacterium tuberculosis*, complicated by congenital factor XIII deficiency. After three months of intercostal drainage placement, the patient developed severe anemia and bleeding tendencies, necessitating a referral to clinical hematology. Genetic testing revealed factor XIII deficiency. This case highlights the complicated interplay between tuberculosis-related complications and a coexisting genetic disorder, highlighting the importance of comprehensive clinical assessment and multidisciplinary management.

## Introduction

Pneumothorax, empyema, and pyopneumothorax are rare complications (prevalence of pneumothorax is 1-2% [1], and that of emphysema is 8.9% [2]) of pulmonary tuberculosis. They contribute significantly to the morbidity and mortality in such complicated cases. As evident by the name, pyopneumothorax is characterized by both pus and gas in the pleural space [3]. Factor XIII deficiency is a rare bleeding disorder with a difficult clinical diagnosis. The usual laboratory investigations, like prothrombin time (PT), international normalized ratio (INR), and activated partial thromboplastin time (aPTT), are within normal limits since factor XIII is not involved in the formation of fibrin. Clinical suspicion should arise in patients presenting with characteristic bleeding, and proper interpretation of laboratory investigations is a must for diagnosing this rare but fatal condition [4].

## Case presentation

**Figure 1:**
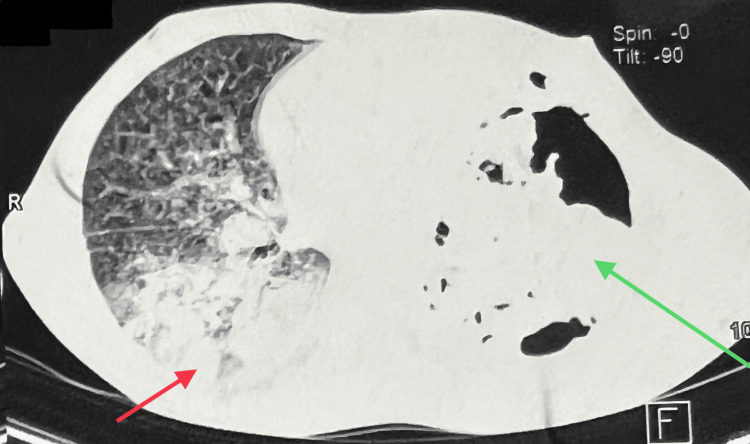
Axial computerized tomography of the thorax in the lung window showing consolidation on the right side (red arrow), and pyopneumothorax with underlying consolidation on the left side (green arrow)

An 18-year-old boy, who was a follow-up case of left-sided pyopneumothorax due to pulmonary tuberculosis, presented to the respiratory medicine outpatient department with complaints of left-sided chest pain and uncontrolled bleeding from the port side. Six months back, he had presented with complaints of shortness of breath and left-sided chest pain for one month. On admission, an acid-fast bacilli smear and cartridge-based nucleic acid amplification test came out to be positive for drug-sensitive *Mycobacterium tuberculosis*. He was started on anti-tubercular treatment. During the course of treatment, he had developed left-sided pyopneumothorax due to pulmonary tuberculosis three months back (Figure 1).

An intercostal drain (ICD) was placed, during which the patient had mild bleeding from the port site, and it was managed by tranexamic acid. The patient had been discharged following that. 

He was born of a non-consanguineous marriage and had been suffering from neuromuscular weakness, hemolytic anemia, recurrent infections, and bleeding disorders since day four of birth. There was also a history of death of two elder siblings due to recurrent infections.

During the current visit, he had completed six months of anti-tubercular treatment (ATT). On examination, he had pallor, his vitals were normal, and all other examination findings were within normal limits. The ICD column was found to be not moving, output was less than 10mL, sediments were absent, and there was no evidence of any bronchopleural fistula formation. His hemoglobin was found to be 3.3g/dL. Due to the low hemoglobin levels and bleeding tendency, he was referred to the Department of Clinical Hematology. Four units of packed red blood cells (RBCs) were transfused, and finally, the hemoglobin increased to 9.1g/dL within two days. He also received eight units of fresh frozen plasma for the bleeding tendency. After twenty days, his laboratory values have been summarized in Table 1.

**Table 1:** Values of patient's laboratory parameters ADP - adenosine diphosphate

Laboratory Parameters	Patient's values	Reference range
Hemoglobin (g/dL)	11.2	13.8-17.2
Total leukocyte count (/cu.mm)	9,000	4,000-11,000
Differential leukocyte count		
Neutrophils (%)	74	55-70
Lymphocytes (%)	20	20-40
Monocytes (%)	4	2-8
Eosinophils (%)	2	1-3
Platelet count (/cu.mm)	370,000	150,000-450,000
General blood picture	Normocytic normochromic RBCs, and platelets of normal morphology, but platelet aggregation was also seen	
Clot retraction (%)	50	48-64
Prothrombin time (s)	13	11-12.5
Activated partial thromboplastin time (s)	34	30-40
Platelet aggregation study with 5mcM ADP (%)	36	69-88
Platelet aggregation study with 1.75mg/mL ristocetin (%)	42	60-180
Urea clot lysis (%)	Partial (80)	50-150

History and laboratory investigations were both suggestive of factor XIII deficiency. DNA testing was done, and the single nucleotide variation (SNV) or indels report has been summarized in Table 2.

**Table 2:** The single nucleotide variation (SNV) or Indels DNA test report of the patient showing factor XIII deficiency

Gene	Location	Variant	Zygosity	Disease	Inheritance	Classification
F13A1 (-) (ENST00000264870.8)	Exon 10	c.1226G>A (p.Ar8409Gin)	Homozygous	Deficiency of factor XIII	Autosomal recessive	Likely pathogenic
ITGA2B (-) (ENST00000262407.6)	Exon 12	c.1018G>A (p.Gly340Ser)	Heterozygous	Platelet-type bleeding disorder-16/ glanzmann thrombasthenia-1	Autosomal dominant/ Autosomal recessive	Uncertain significance

No significant copy number variants were found. The reports, along with the history, were suggestive of a congenital factor XIII deficiency.

The patient has been responding well to the ATT and is currently asymptomatic. Hence, no surgical intervention is required.

## Discussion

Here, we report a patient with pyopneumothorax, secondary to tuberculosis, with superadded factor XIII deficiency, which further complicated the hospital stay and interventions due to increased bleeding tendencies.

Pneumothorax, empyema, and pyopneumothorax develop as a result of the rupture of mycobacterial pulmonary foci into the pleural space, setting up hypersensitivity reactions. The pleural capillaries' permeability to protein increases due to delayed hypersensitivity reactions. An increase in the protein content of the pleural fluid stimulates a greater rate of pleural fluid production. A high protein concentration can impede the pleural lymphatic system, lowering the rate at which the fluid is cleared. Pleural fluid hence builds up as a result of an increase in pleural fluid generation and a reduction in fluid drainage [5]. An alveolar or bronchopleural fistula that results in subsequent pneumothorax and empyema can be caused by tuberculous pleural effusion in combination with severe lung parenchymal abnormalities [6]. Tuberculous empyema represents a chronic, active infection of the pleural space that contains a large number of tubercle bacilli. Long-standing tuberculous empyema leads to pleural thickening and even calcification. The pleural effusion in such cases is neutrophil-predominant, with a high mycobacterial load [7]. Acid-fast bacilli smears have a better predictive value in diagnosing tuberculous empyema than tuberculous pleural effusion [8]. Empyema and pyopneumothorax are often caused by insufficient or ineffective anti-tubercular therapy, in which the mycobacteria become resistant, or the medications don't reach the mycobacteria well enough because fibrin deposits have thickened the pleura [9]. 

The a-subunit of factor XIII is synthesized by cells of monocyte lineage, like macrophages and histiocytes. So, other than clot retraction, factor XIIIa (FXIIIa) also has significant roles in inflammatory processes. In a study by Probst-Cousin et al. in 1997, FXIIIa expression was studied in granulomatous lesions of sarcoidosis and mycobacterial infection. FXIIIa-positive macrophages were predominant in the periphery of the granulomas, but these were absent in the centers. The study concluded that FXIIIa-producing macrophages can be hypothesized to play a role in centripetal fibrosis in granulomatous inflammation [10].

Patients with FXIII deficiency quite often suffer from recurrent infections and have suppressed immunity, as was also seen in our patients. The role of FXIII in wound healing is well-known from preclinical studies. In FXIII-deficient mice, excisional wound healing was found to be delayed compared to controls or FXIII-deficient mice receiving FXIII supplementation. Moreover, FXIII cross-links fibrin to surface proteins of invading bacteria (e.g., *Streptococcus, Staphylococcus, *and* Escherichia*), trapping bacteria in the clot and reducing the risk of tissue infection. Hence, a deficiency of factor XIII increases the risk of infection [11].

Treatment options for people with factor XIII deficiency are numerous. The product that is most frequently administered is a cryoprecipitate, which contains 20% to 30% of the original factor XIII of plasma [12]. Even fresh frozen plasma can be administered in acutely bleeding patients [13]. In 2011, the FDA approved the use of virus-inactivated FXIII concentrate made from human plasma for perioperative treatment and prevention of patients with congenital FXIII deficiency [14]. Another item, catridecacog, a recombinant FXIII-A (rFXIIIA) subunit, received FDA approval in 2013 [15].

## Conclusions

This was a case where two rare entities, i.e., pyopneumothorax due to tuberculosis and a congenital factor XIII deficiency, manifested in the same patient, hence making the management far more challenging. Having a high degree of suspicion is important in patients presenting with uncontrollable bleeding, and getting the appropriate laboratory investigations done and interpreting them in the right direction is a must for diagnosing this rare and fatal disease.
